# Onchocerciasis: Target product profiles of in vitro diagnostics to support onchocerciasis elimination mapping and mass drug administration stopping decisions

**DOI:** 10.1371/journal.pntd.0010682

**Published:** 2022-08-03

**Authors:** Marco A. Biamonte, Paul T. Cantey, Yaya I. Coulibaly, Katherine M. Gass, Louise C. Hamill, Christopher Hanna, Patrick J. Lammie, Joseph Kamgno, Thomas B. Nutman, David W. Oguttu, Dieudonné P. Sankara, Wilma A. Stolk, Thomas R. Unnasch

**Affiliations:** 1 Drugs & Diagnostics for Tropical Diseases, San Diego, California, United States of America; 2 Division of Parasitic Diseases and Malaria, U.S. Centers for Disease Control and Prevention, Atlanta, Georgia, United States of America; 3 Mali International Center for Excellence in Research, Faculty of Medicine and Odonto-Stomatology, University of Sciences, Techniques and Technologies of Bamako, Bamako, Mali, Dermatology Hospital of Bamako, Bamako, Mali; 4 Neglected Tropical Diseases Support Center, Task Force for Global Health, Decatur, Georgia, United States of America; 5 Sightsavers, Haywards Heath, United Kingdom; 6 Global Project Partners, Oakland, California, United States of America; 7 Centre for Research on Filariasis and other Tropical Diseases, Yaoundé, Cameroon, Faculty of Medicine and Biomedical Sciences, University of Yaoundé I, Yaoundé, Cameroon; 8 Laboratory of Parasitic Diseases, National Institute of Allergy and Infectious Diseases, Bethesda, Maryland, United States of America; 9 Vector Control Division, Ministry of Health, Kampala, Uganda; 10 Department of Control of Neglected Tropical Diseases, World Health Organization, Geneva, Switzerland; 11 Department of Public Health, Erasmus MC, University Medical Center Rotterdam, Rotterdam, the Netherlands; 12 Global Health Infectious Disease Research Program, University of South Florida, Tampa, Florida, United States of America; Imperial College London, Faculty of Medicine, School of Public Health, UNITED KINGDOM

## Abstract

In June 2021, the World Health Organization (WHO), recognizing the need for new diagnostics to support the control and elimination of onchocerciasis, published the target product profiles (TPPs) of new tests that would support the two most immediate needs: (a) mapping onchocerciasis in areas of low prevalence and (b) deciding when to stop mass drug administration programs. In both instances, the test should ideally detect an antigen specific for live, adult *O*. *volvulus* female worms. The preferred format is a field-deployable rapid test. For mapping, the test needs to be ≥ 60% sensitive and ≥ 99.8% specific, while to support stopping decisions, the test must be ≥ 89% sensitive and ≥ 99.8% specific. The requirement for extremely high specificity is dictated by the need to detect with sufficient statistical confidence the low seroprevalence threshold set by WHO. Surveys designed to detect a 1–2% prevalence of a given biomarker, as is the case here, cannot tolerate more than 0.2% of false-positives. Otherwise, the background noise would drown out the signal. It is recognized that reaching and demonstrating such a stringent specificity criterion will be challenging, but test developers can expect to be assisted by national governments and implementing partners for adequately powered field validation.

## Author summary

River blindness, also known as onchocerciasis, affects 21 million people, predominantly in sub-Saharan Africa. For decades, the international community has fought this disease through mass drug administration (MDA) programs focused on controlling morbidity in areas of high prevalence. Now, as part of their 2021–2030 Roadmap for Neglected Tropical Diseases, the World Health Organization (WHO) has set an ambitious goal, shifting from controlling to eliminating onchocerciasis. This implies addressing areas of low infection prevalence. As a result, new diagnostics tools are required to identify and map areas of low onchocerciasis prevalence and to help decide where to initiate MDA. Similarly, new diagnostics are required to decide when the prevalence of onchocerciasis is sufficiently low to justify stopping MDA. A WHO-appointed independent panel, the Diagnostics Technical Advisory Group for Neglected Tropical Diseases, and its subgroup specific to onchocerciasis, have established the desired Target Product Profiles (TPPs) for such new tests. The TPPs were posted in June 2021 on the WHO website. This article describes the methodology used to produce the TPPs, with an emphasis on calculating the required sensitivity and specificity characteristics.

## Introduction

**Fig 1:**
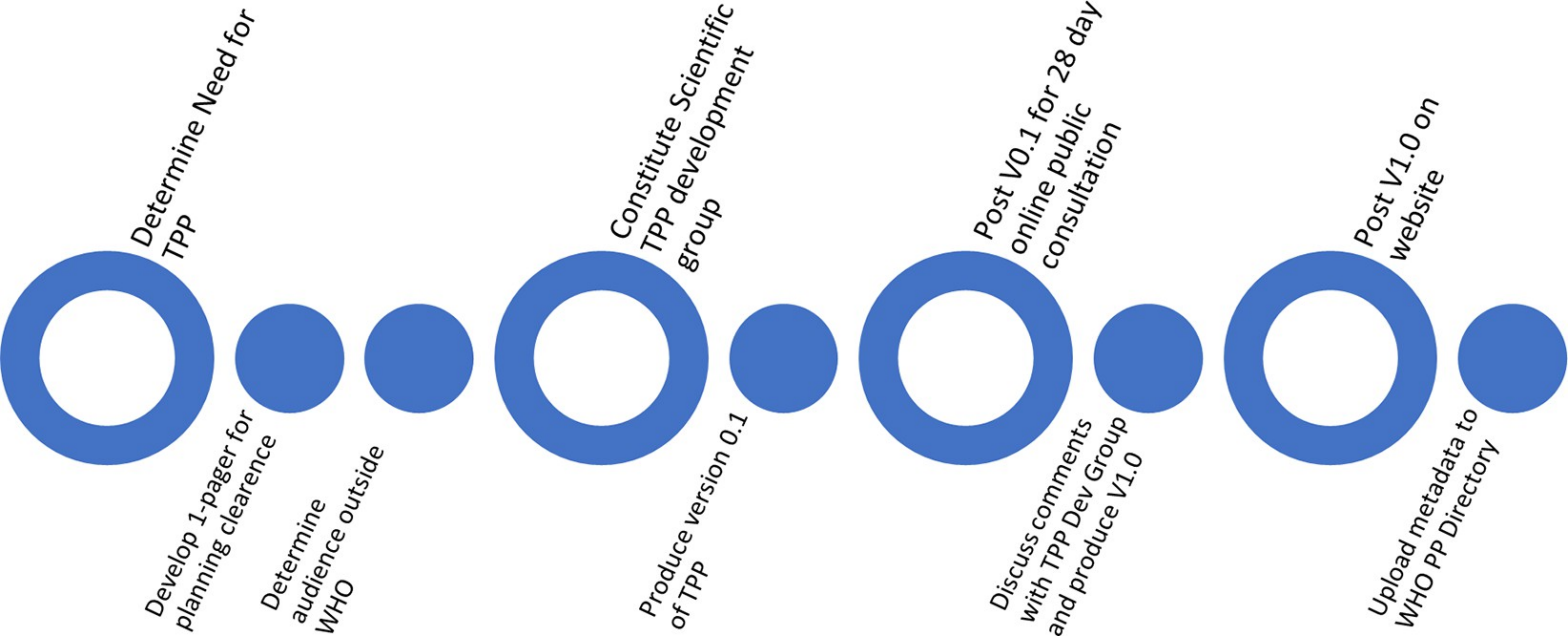
World Health Organization process to establish new target product profiles [8]. PP = product profile.

The Department of Control of Neglected Tropical Diseases at the World Health Organization (WHO) has established a roadmap to the 2030 goals for neglected tropical diseases (NTDs) [1]. This document highlights new diagnostics as one of the critical needs to achieve the desired goals. In 2019, the same department assembled the Diagnostic Technical Advisory Group for NTDs (DTAG-NTD), which oversees the work of several subgroups organized by disease area and involving additional domain experts [2]. Each disease subgroup was tasked with drafting the target product profiles (TPPs) of diagnostics that are essential to meeting the 2030 goals. Since the establishment of the DTAG, TPPs have been published for lymphatic filariasis [3, 4], onchocerciasis [5], schistosomiasis [6], soil-transmitted helminthiases [7], and additional draft TPPs for other NTDs are in the pipeline. These TPPs were generated using the WHO process summarized in **Fig 1** [8].

The onchocerciasis DTAG subgroup consists of the authors of this article, who met online in the Summer and Fall of 2020 in the presence of observers representing donor agencies, major nonprofit groups, and WHO representatives. Priorities of the onchocerciasis subgroup were to establish TPPs for tests that will support (a) the mapping of onchocerciasis in areas of low prevalence, and (b) the decision to stop mass drug administration (MDA) programs. This article describes the background and methodology that led to the published TPPs, with an emphasis on sensitivity and specificity criteria.

## Epidemiology

Onchocerciasis, also known as river blindness, affected an estimated 21 million people in 2019, with 99% of the cases in 31 sub-Saharan countries [9, 10]. The disease is caused by the filarial worm *Onchocerca volvulus*, which is transmitted by *Simulium* blackflies. Adult worms live in nodules, some of which are subcutaneous. In contrast, the first stage larvae (microfilariae) migrate through the skin, causing debilitating pruritus and skin diseases, and to the eyes, leading to progressive and irreversible blindness. Onchocerciasis is also hypothesized to lead to neurological disorders, including epilepsy [11], nodding syndrome [12], and stunted growth [13].

## Public health response

Currently, at least 218 million people live in areas known to be endemic for onchocerciasis [14]. Morbidity is controlled by annual mass drug administration of ivermectin (Mectizan®), with 154 million people receiving medication in 2019 [14]. This drug temporarily blocks infection transmission by clearing microfilariae from human skin, but it does not kill the adult worms, which have a reproductive lifespan of 10 or more years, requiring MDA to be continued for many years. Ivermectin is donated to endemic countries “as much as needed, for as long as needed” by the pharmaceutical company Merck and Co., Inc. Other registered drugs such as moxidectin and doxycycline, and new macrofilaricidal clinical candidates, may, in the future, help to accelerate elimination efforts [15–17].

In 2010, based on progress towards elimination of transmission of onchocerciasis in the Americas as well as in a few foci in sub-Saharan Africa, global targets were revised from the control of morbidity to elimination (interruption of transmission) [18]. In 2021, following several rounds of public consultation and an endorsement by the 73^rd^ World Health Assembly, WHO published a “road map for neglected tropical diseases 2021–2030” [1]. In this document, the principal disease-specific target for onchocerciasis is to increase the number of countries verified as having interrupted transmission from 4 (12%, Colombia, Ecuador, Guatemala, Mexico) in 2020 to 12 (31%) in 2030. To reach this goal, the road map identifies as a critical action the mapping of suspected onchocerciasis- endemic areas, with subsequent initiation of MDA wherever indicated. It further identifies as critical the development of improved diagnostics to facilitate mapping and decision-making [1].

The shift from control to elimination requires treatment be expanded to areas classified as hypo-endemic and that were consequently not targeted for MDA originally. Hypo-endemic areas were defined in 1979 as having less than 35% prevalence of microfilarial infection and less than 1% of blindness [19]. Subsequently, during the Rapid Epidemiological Mapping for Onchocerciasis (REMO) [20, 21], implemented by the African Programme for Onchocerciasis Control (APOC) between 1997–2007, a 20% prevalence of nodules as detected by palpation was correlated with risk of blindness in a community [22] and areas below that threshold were not treated. It was estimated that in 2011, 77 million resided in areas with 5–20% nodule prevalence [23].

The REMO surveys, however, were neither designed to nor capable of detecting low infection prevalence in the 1–2% range [24], as deemed required for elimination. In addition, the maps are not current and with few exceptions have not been re-surveyed using the more recent serological or entomological techniques. This causes uncertainty in the total number of people who must be reached. Assessment or reassessment is needed in many of these areas to decide where to start MDA, while sensitive diagnostics are needed to inform stopping decisions for the 218 million people currently living in areas where MDA is ongoing.

## Available diagnostic tools and their limitations

The main diagnostics for onchocerciasis are:

1. Analysis of skin-biopsies (skin snips) by microscopy or molecular techniques. This method is considered to be definitive but is relatively insensitive in areas of low infection prevalence and even in areas of high prevalence after several rounds of MDA. Skin snips can be painful for the patient, more so if appropriate equipment and technique are not ensured, and the technique has low throughput. Furthermore, populations are reluctant to participate in skin snipping, especially when onchocerciasis is not viewed by residents as a problem and/or when children are involved. One caveat is that *L*. *loa* microfilariae can be found in skin snips of people with high *L*. *loa* microfilarial densities [25].
2. Nodule palpation has been a main driver of APOC and has been used for the Rapid Epidemiological Assessment (REA)/Rapid Epidemiological Mapping of Onchocerciasis. A prevalence of approximately 5% of people having palpable nodules of other etiologies makes this technique acceptable to identify meso- and hyper-endemic areas but palpation is insufficiently specific for hypo-endemic areas [24]. Furthermore, deep-tissue nodules are not palpable, adding a problem of sensitivity.
3. Worm movement in onchocercal nodules can be detected by ultra-sound (ultrasonography) and while this technique can be applied for case management and in clinical trials, it is too difficult to deploy for mapping efforts [26].
4. The DEC patch is a diethylcarbamazine-containing transdermal patch that kills microfilariae in the skin, triggering a reactive urticaria that can be visualized (Mazzotti reaction). This technique requires a total of two days in the field, one to apply the patch and a second one to monitor skin reactions, which represents a practical limitation. Additionally, specificity issues arise in areas co-endemic with *L*. *loa* [27]. “Ready-to-use” DEC patches made under Good Manufacturing Procedures (GMP) conditions by a manufacturer specialized in transdermal-delivery systems are available to ministries of health requesting them from WHO (TDR contact: Dr. A. C. Kuesel) [28]. To date, large scale evaluation of the DEC patch has only occurred in populations that were skin snip negative [29]. The evaluation now needs to be conducted in populations with different levels of skin microfilarial density to assess its performance and safety.
5. Ov16 serology is part of the current WHO criteria for stopping MDA, alongside entomological investigations [30]. It is also recommended to identify hypo-endemic areas. There are three ELISAs, of which one was commercial and is now discontinued (BIOLINE Onchocerciasis IgG4 ELISA, Abbott), one was developed at the Centers for Disease Control and Prevention (CDC) [31], and one was developed by the Onchocerciasis Elimination Program for the Americas (OEPA) [32]. There is also one commercial rapid test (BIOLINE Onchocerciasis IgG4 Rapid Test, Abbott). The Report of the 3^rd^ Meeting of the WHO Onchocerciasis Technical Advisory Subgroup (OTS), a body unrelated to the DTAG-NTD, summarized the results of the evaluation of different Ov16 assays in a variety of programmatic contexts and identified differences in performance with different types of specimens and identified concerns of accuracy [33]. The lack of standardization across different versions of the same serological test complicates decision-making. The requirement for cold-chain shipping for commercial ELISA kits, or components needed for OEPA or CDC ELISA protocols is a further logistical complication.
6. Entomological identification of ongoing transmission consists of detecting infective or infected *Simulium* flies by PCR using primers for the O-150 repeated sequence specific to *O*. *volvulus*. Blackfly heads are processed in pools of between 50–100; processing thoraxes and bodies can also yield useful information on vector-parasite contact. PCR requires trained laboratory personnel and specialized equipment and reagents. Blackfly collections require field teams knowledgeable about methodologies for finding and capturing flies. Most programmatic *Simulium* surveys utilize ethically concerning and resource intensive human landing capture.

Of the above 6 techniques, skin biopsies, nodule palpation, ultrasonography, and DEC-patch are meant to detect onchocerciasis at the individual level, and Ov16 serology and entomology at the population level.

## Need for novel target product profiles

With over 150 million doses of ivermectin distributed each year, the fight against onchocerciasis is a major international public health program. Diagnostics are required at different phases of the program, namely during: (1) mapping, (2) monitoring and evaluation of MDA programs, (3) decision to stop MDA, (4) post-treatment surveillance, and (5) post-elimination surveillance.

### Mapping

A sensitive and specific assay is required to map onchocerciasis in hypo-endemic areas and to overcome the limitations of the existing arsenal. The WHO Onchocerciasis Technical Subgroup recommended establishing a biological threshold of 2% antibody prevalence in adults for decisions to start treatment [33]. Field-based operational research to generate empirical data to support the 2% starting threshold is underway. To have confidence of estimates around this threshold, sensitive and specific tests are critical, and the corresponding TPP is described in this article.

### Monitoring and Evaluation (M&E)

There are no formal WHO guidelines for monitoring and evaluation. In areas of higher endemicity, MDA progress towards interruption of transmission is evaluated with skin snips (microscopy or PCR) or using serology. Given the long-lasting nature of the Ov16 response, serology cannot be performed in adults to monitor a decrease in transmission. Hence, serology is performed in children as sentinel groups. Monitoring of infected or infective vectors is appropriate and recommended by WHO but has never been operationalized due to challenges of logistics and costs. Unlike mapping, which is done only once, monitoring must be continued throughout the life of the program, and therefore the new tools need to be especially affordable. A TPP for Monitoring and Evaluation was not addressed by the onchocerciasis DTAG subgroup.

### Stopping MDA

Stopping should be a one-time event, given the difficulty in restarting a program after it has been stopped and the risk of resurgence of transmission of stopping prematurely. One may justifiably use more resources or more expensive tests for the sake of supporting appropriate decision-making on MDA cessation. In 2016, WHO published guidelines for stopping mass drug administration and verifying elimination of human onchocerciasis [30]. The guidelines require that both entomology and serology be used to demonstrate the interruption of transmission of *O*. *volvulus* for the purpose of stopping MDA.

- Entomology (“strong recommendation, high certainty of evidence”): O-150 PCR (Poolscreen) testing in black flies should be used to demonstrate interruption of transmission of *O*. *volvulus*, with an upper bound of the 95% confidence interval of a prevalence of vectors carrying infective larvae of less than 0.05%. Research priorities for entomological evaluations include standardizing the PCR diagnostic test itself and the protocols for fly collection.
- Ov16 serology (“strong recommendation, low certainty of evidence”): Ov16 ELISA should meet a threshold of 0.1% positivity in children at risk younger than 10 years old (the upper bound 95% confidence interval should exclude 0.1%). Research priorities identified in the WHO guidelines also include investigating the sero-reversion of Ov16 responses and validating the Ov16 RDT.

Dspite these guidelines, the serological threshold of 0.1% positive rate is now considered to be too stringent and probably impossible to measure accurately given the specificity of the existing tests [33]. Furthermore, evidence to support this threshold and age-group is low and research is ongoing to identify the most informative age groups and define associated thresholds in different epidemiological settings [34, 35]. Operational research is needed to determine and validate a new, appropriate serological stopping threshold. In the meantime, the OTS has tentatively proposed a 1% threshold, and our subgroup therefore established a TPP on the basis of this hypothetical, conservative 1% threshold.

### Post-treatment surveillance (PTS)

Entomological surveys are conducted beginning 12 months after the last round of MDA and during peak transmission season. Following a PTS period of 3–5 years, and on the advice of the national onchocerciasis expert elimination committee, interruption of transmission is confirmed by entomological test (O-150 PCR Poolscreen) and if necessary, by additional serological testing (Ov16). We are not providing a TPP for post-treatment surveillance.

### Post-elimination surveillance (PES)

After WHO grants elimination status, a national program establishes a PES system to detect possible renewal of parasite transmission (resurgence or reintroduction) both in previously endemic and in non-endemic areas as well as in areas where imported cases might be expected to occur. Such PES might be centered on entomological assessments and testing of caught flies by O-150 PCR. Such assessments should be conducted at regular intervals until elimination is verified in all countries in the relevant WHO region, or at least until any risk of resurgence or reintroduction can substantially be excluded. It is likely that in a post-elimination surveillance setting, few resources will be available, and entomology may be too expensive and technically demanding to be routinely performed. Therefore, it would be desirable to have a diagnostic that can be easily integrated within routine health system activities. With many countries still being far from interruption of transmission, diagnostics specifically designed for post-elimination surveillance are regarded as a lower priority at this time and a corresponding TPP was not addressed.

### Individual diagnosis and case management

Eventually, there will be a need outside of WHO’s preventative chemotherapy approach to onchocerciasis. A diagnostic that enables individual case detection and management will have to identify individuals carrying fecund adult female parasites, but such a TPP was not considered by our subgroup.

## Summary of TPPs

The TPPs of diagnostics to map onchocerciasis and to decide when to stop MDA are available on the WHO website [5]. **Tables 1** and **2** summarize the TPPs for mapping and stopping, respectively; the reader is referred to the published TPPs for all 41 criteria [5].

**Table 1:** Selected features of the target product profile of a diagnostic to map onchocerciasis in low prevalence areas. Criteria that are generally expected of a rapid diagnostic test for a NTD are not included in this summary. For all 41 criteria, please see the original publication [5]. The word “biomarker” includes an antibody.

1. Product use summary	Ideal	Minimum
1.1 Intended use	An *in vitro* point-of-care test to map onchocerciasis and identify areas with >2% prevalence of analyte(s).	An *in vitro* laboratory-based test to map onchocerciasis and identify areas with >2% prevalence of analyte(s).
1.2 Targeted population	All ages of individuals resident in the population living in the defined geographic area.	Sentinel groups of school-age children living in the defined geographic area.
1.3 Lowest infrastructure level	The test will be performed under "zero-infrastructure" conditions including but not limited to community health centers, households, and outdoor conditions.	If the required levels of performance necessitate a laboratory-based test, tests can be performed in a centralized laboratory.
**2. Design**	**Ideal**	**Minimum**
2.8 Target analyte	Antigen(s) or other biomarker(s) specific for live, adult *O*. *volvulus* female worms	Biomarker(s) to detect exposure to *O*. *volvulus*.
2.9 Type of analysis	Quantitative	Qualitative
**3. Performance**	**Ideal**	**Minimum**
3.1 Species differentiation	Can differentiate *O*. *volvulus* from *Wuchereria*, *Loa*, and *Mansonella* spp.	Same
3.2 Diagnostic/clinical sensitivity	≥ 60%	Same
3.3 Diagnostic/clinical specificity	≥ 99.8%	Same
3.7. Target shelf life/stability	Stable for 36 months at 4–40°C. Tolerates excursions to 50°C for 2 weeks	If laboratory based: ≥12 months at 4 C; temperature excursion of 50 C for one week acceptable. If field deployable: Stable for 18 months at 4–37°C, Tolerates excursions to 50 C for 1 week.
**5. Product cost and channels**	**Ideal**	**Minimum**
5.1 Target pricing per test	Mapping: < $1.00	Mapping: < $2.50

**Table 2:** Selected features of the target product profile of a diagnostic to support an MDA stopping decision. Criteria that are generally expected of a rapid diagnostic test for a NTD are not included in this summary. For all 41 criteria, please see the original publication [5]. The word “biomarker” includes an antibody.

1. Product use summary	Ideal	Minimum
1.1 Intended use	An *in vitro* point-of-care test to support MDA stopping decision and certify areas with <1% prevalence of analyte(s).	An *in vitro* laboratory-based test to support MDA stopping decision and certify areas with <1% of analyte(s).
1.2 Targeted population	All ages of individuals resident in the population living in the defined geographic area.	Sentinel groups of school-age children living in the defined geographic area.
1.3 Lowest infrastructure level	The test will be performed under "zero-infrastructure" conditions including but not limited to community health centers, households, and outdoor conditions.	If the required levels of performance necessitate a laboratory-based test, tests can be performed in a centralized laboratory.
**2. Design**	**Ideal**	**Minimum**
2.8 Target analyte	Antigen(s) or other biomarker(s) specific for live, adult *O*. *volvulus* female worms	Biomarker(s) to detect exposure to *O*. *volvulus*.
2.9 Type of analysis	Quantitative	Qualitative
**3. Performance**	**Ideal**	**Minimum**
3.1 Species differentiation	Can differentiate *O*. *volvulus* from *Wuchereria*, *Loa*, and *Mansonella* spp.	Same
3.2 Diagnostic/clinical sensitivity	≥ 89%	Same
3.3 Diagnostic/clinical specificity	≥ 99.8%	Same
3.7. Target shelf life/stability	Stable for 36 months at 4–40°C. Tolerates excursions to 50°C for 2 weeks	If laboratory based: ≥12 months at 4 C; temperature excursion of 50 C for one week acceptable. If field deployable: Stable for 18 months at 4–37°C, Tolerates excursions to 50 C for 1 week.
**5. Product cost and channels**	**Ideal**	**Minimum**
5.1 Target pricing per test	< $2.00	< $3.00

The TPPs for mapping and stopping are identical to each other in 38 of the 41 parameters. For both intended uses, the test should ideally detect a biomarker specific for live, adult *O*. *volvulus* female worms, though antibody tests are acceptable. The preferred format is a field-deployable rapid test, but a laboratory test is acceptable in the absence of viable alternatives.

The key difference between the two TPPs is the prevalence of the analyte that must be detected, which impacts the targeted sensitivity. For mapping, the test needs to detect as little as 2% prevalence of the analyte under scrutiny and therefore be > 60% sensitive and > 99.8% specific. For stopping decisions, the test must detect as little as 1% of analyte prevalence, and consequently the test must be > 89% sensitive and > 99.8% specific. The methodology used to derive those numbers is explained in the discussion. The only other difference is the cost, which must be less than $2.50 per test for mapping and $3.00 for stopping MDA.

## General comments on the TPP criteria

The above-mentioned TPPs attempt to strike a balance between ideal and pragmatic scenarios. All 41 criteria [5] were weighed and deliberated with accessibility, ease of use, and robustness in mind, as well as the urgency of the current need for new diagnostics to help achieve WHO 2030 elimination goals. For instance, the ideal intended use is at the point of care, in zero infrastructure settings. However, laboratory-based diagnostics are acceptable since they are likely to have a more rapid development path. Similarly, identifying an antigen indicative of the presence of live female *O*. *volvulus* worms is ideal, but probably extremely difficult, and therefore serology tests are acceptable even if suboptimal because they will be more rapidly developed. Across many of the 41 criteria, even the minimum acceptable characteristics will prove challenging for assay developers. For example, the target price per test is not likely to be achieved without significant support from external donors and multilateral organizations, as has been seen for previous onchocerciasis diagnostics, and more recently for COVID-19 rapid tests for low- and middle-income countries [31]. The sensitivity and specificity criteria will prove perhaps the most challenging to meet, particularly in an ideal-case zero-infrastructure environment. The rationale for setting such stringent targets for these key criteria is explained in depth below.

Across the 13 design criteria, quality, sensitivity, and specificity were key, viewed through the lens of the ideal intended use case. While laboratory capacity in Sub-Saharan Africa is strong in many countries and disease specialties, dedicated NTD laboratory facilities are still relatively uncommon, and frequently under-resourced where they do exist. This was another key factor during the deliberations of the DTAG group and should remain a key consideration for potential assay developers. On the positive side, there is an enormous amount of political will and in-country expertise directed towards onchocerciasis elimination. The achievements that have been made to date with the available sub-optimal techniques are remarkable. Through these TPPs, this progress towards elimination can be accelerated.

## Methods

### Surveys and associated parameters: Key concepts

Particular attention was devoted to setting the sensitivity and specificity requirements for new tests. It must be stressed that the diagnostics will not be used for individual case management, but in the context of surveys–most likely population-based cluster surveys. The fundamental concepts of surveys are analogous to those of diagnostic tests. In one case, one interrogates a population, in the other one interrogates an individual, asking if the disease is present. For both individual treatment and population surveys, one wishes to provide a correct positive answer when the disease is truly present, as well as a correct negative answer when the disease is truly absent.

The performance of a diagnostic test for individual case management is summarized in its sensitivity and specificity. A sensitivity of 95% means that the test detects 95% of the individuals who truly have the infection (5% of those infected will give a falsely negative test). Conversely, a specificity of 90% means that the test correctly identifies as negatives 90% of those who do not have the infection (10% of those without infection will give a falsely positive test). Similarly, in a survey, one may want to detect 95% of the areas in which *O*. *volvulus* transmission is ongoing (and accept that 5% of the infected populations will falsely test as onchocerciasis-free). Conversely, one may want to correctly identify as negatives 90% of the areas where *O*. *volvulus* transmission is truly absent or non-sustainable (and accept that 10% of the infection-free populations will falsely test as onchocerciasis endemic). Such a survey, if used to decide when to start MDA programs, would lead to 5% of under-treated areas, and 10% of over-treated areas.

The tolerance for 5% under-treatment and 10% of over-treatment are precisely the figures that were selected for the onchocerciasis programs. In technical terms, these figures are known as Type I error (α) and Type II error (β), respectively. The Type I error is defined as the risk of a false-negative survey outcome (5% in the example above) while the Type II error is the risk of a false-positive survey outcome (10% in the example above).

The power of a survey is defined as 1 – β, and for the mapping and stopping TPPs is 90%. Power and specificity are related concepts, in that they both measure the aptitude of a survey/test to correctly identify as negative populations or individuals that are not diseased. However, the two parameters are not identical. A test with 99% specificity has, by definition, a 1% chance of giving a false-positive when tested on a single healthy subject. But when this test becomes part of a village survey and is repeated on 50 healthy participants, the chances of seeing at least one false-positive test increase dramatically. If one were to start MDA whenever a 99% specific test returns 1 or more positive readouts out of a total of 50 people tested, one would erroneously treat as many as 39.5% of the healthy communities (Type II error = 39.5%, power = 60.5%), a far cry from the targeted tolerance of up to 10% overtreatment (Type II error = 10%, power = 90%). The 39.5% figure derives from the fact that the probability that a 99% specific test, when used 50 times in a truly non-infected population, would only return negative results is (0.99)^50^ = 0.605 and therefore the probability that at least one test is falsely positive is 100%– 60.5% = 39.5%.

The Type I and II errors are affected by additional parameters: the biological threshold, the critical cutoff, and the sample size. The biological threshold is defined as the prevalence above which onchocerciasis transmission becomes self-sustainable. The currently assumed biological threshold for initiating mass treatment is 2% antibody prevalence in adults; however, this threshold is intentionally conservative and is likely to be increased as additional insights from empiric field evidence and models become available.

The critical cutoff represents the number of positive tests at or above which the survey area is classified as “positive” (meaning exceeding the biological threshold); survey areas falling below the critical cutoff are classified as “negative”. In the previous example, the critical cutoff was ≥ 1 positive test in a cluster. Increasing the critical cutoff improves the power of a survey but also increases the Type I error. This is akin to increasing the cutoff of an ELISA, which improves specificity at the expense of sensitivity.

## Results

### Establishing the required sensitivity and specificity for onchocerciasis elimination mapping

The OTS has proposed a strategy for onchocerciasis elimination mapping (OEM) [33, 36] in two parts:

- Stage 1: Purposeful sampling. Five first-line villages are selected based on a potentially high risk of transmission, with a convenience sample of 100 adults/village (first-line villages are usually those closest to a vector breeding site, with some exceptions related to the local environment). If the Ov16 prevalence exceeds the biological threshold of 2% in at least 1 village, then MDA should be initiated. If the threshold is not exceeded then additional sampling to check for any overlooked areas of transmission is recommended, but the exact approach for undertaking stage 2 sampling is a provisional recommendation. Conducting stage 1 purposeful sampling is a strong recommendation based on robust evidence and unlikely to change. Adjustment of the threshold in light of new or improved diagnostics is likely.
- Stage 2: Systematic random sampling. Twenty villages are systematically selected from the list of all villages in the survey area, with a sample of 50 adults/village. If the prevalence exceeds the biological threshold of 2% in at least 1 village, then MDA should be initiated. If not, the area does not need MDA.

The onchocerciasis DTAG subgroup proceeded by first determining the required specificity, followed by the sensitivity. The first task was to calculate the minimum specificity needed to prevent unnecessary MDAs ≥ 90% of the time. This is equivalent to selecting a power of ≥ 90% and a Type II error of < 10%.

The effect of the specificity on the Type II error was calculated (**Table 3**) using the R statistical software (version 3.4.4). Calculations assumed a survey of 20 villages, with 50 participants per village, and no endemic onchocerciasis. The critical cutoff to consider the survey positive was set at ≥ 2 positive tests in at least 1 village. To achieve a Type II error below 10%, a specificity of 99.8% was required (Table 3, entry 5).

**Table 3:** Effect of specificity on the Type II error of a cluster survey involving 20 villages, and 50 people/village. No sensitivity assumptions were made. The critical cutoff was ≥ 2 positive tests in at least 1 village. The calculations were made for a true prevalence of 0%. At least 99.8% specificity is required to reach a Type II error of < 10%.

Entry	Sensitivity	Specificity	True Prevalence	Critical cutoff	Type II error
1	NA	99.0%	0%	≥ 2 positives	84.6%
2	NA	99.5%	0%	≥ 2 positives	41.1%
3	NA	99.6%	0%	≥ 2 positives	29.5%
4	NA	99.7%	0%	≥ 2 positives	18.3%
5	NA	99.8%	0%	≥ 2 positives	8.8%
6	NA	99.9%	0%	≥ 2 positives	2.3%

Having established the specificity requirement, the subgroup examined the minimum sensitivity needed to capture ≥ 95% of the areas that require MDA. In other words, the survey needs to correctly identify areas of 2% prevalence with a Type I error < 5%. The calculations reported in **Table 4** indicate that the test sensitivity must be at least 60% sensitive.

**Table 4:** Effect of sensitivity on the Type I error of a cluster survey involving 20 villages, and 50 people/participant. A specificity of 99.8% was assumed. The critical cutoff was ≥ 2 positive tests in at least 1 village. The calculations were made for a true prevalence of 2%. At least 60% sensitivity is required to reach a Type I error of < 5%.

Entry	Sensitivity	Specificity	True Prevalence	Critical cutoff	Type I error
1	100%	99.8%	2%	≥ 2 positives	0.08%
2	90%	99.8%	2%	≥ 2 positives	0.22%
3	80%	99.8%	2%	≥ 2 positives	0.59%
4	70%	99.8%	2%	≥ 2 positives	1.48%
5	60%	99.8%	2%	≥ 2 positives	3.50%
6	50%	99.8%	2%	≥ 2 positives	7.68%

### Establishing the required sensitivity and specificity to support stopping decision

The WHO guidelines to stop onchocerciasis treatment are to target a 0.1% true seroprevalence of Ov16 antibodies detected by ELISA, and 0.1% L3 larvae DNA in the heads of parous black fly vectors [30]. The evidence base for the serological threshold of 0.1% was, and remains weak, but was deliberately conservative to minimize premature cessation of MDA. Many consider it too conservative; we developed our TPP based on a hypothetical 1% biological threshold. The WHO 2016 guidance further recommends basing decisions on an average survey area prevalence, based on the results of a cluster survey. However, such an approach may mask any heterogeneity within the survey area, causing programs to miss hotspots that merit further treatment. If the same logic as for the onchocerciasis elimination mapping (OEM) holds, future surveys design will require cluster-level decision making. Here, two scenarios for stop MDA decisions were considered based on: 1) the average prevalence from a 30-cluster survey with a total sample size of 3000; and 2) the cluster-specific results from a survey of the same size and assuming 100 children per cluster. The same process as described for mapping was applied, calculating first the specificity and then the sensitivity (**Box 1**).

**Box 1. Summary of the assumptions and process used to establish the sensitivity and specificity criteria for a test that will support onchocerciasis elimination mapping or MDA stopping decisions**

**1. Assumptions**

**1.1 Mapping**

- MDA should be initiated in areas where the biomarker prevalence in resident adults is 2% or more.
- Five 1st line villages are surveyed with 100 adults/village (Stage 1). If Ov16 prevalence > 2% in at least 1 village, then MDA should be initiated. If not, proceed to Stage 2 by surveying 20 clusters (*i*.*e*., villages) x 50 adults/cluster.
- When ≥ 2 positive tests are obtained within at least one cluster, the entire area will be considered endemic.

**1.2 Stopping**

- MDA should be stopped in areas where the biomarker prevalence in children is 1% or less.
- Assumption: an area will be surveyed by examining 30 clusters (*i*.*e*., villages) x 100 children/cluster (we acknowledge the limitation that in practice not all stop MDA surveys may include 30 x 100 children).
- When ≥ 3 positive tests are obtained within the same cluster, onchocerciasis will be considered still endemic.

                              ⇩

**2. Establish acceptable Type II error**

Accept that 10% of surveys will suggest that onchocerciasis transmission is ongoing, when in fact onchocerciasis is either absent or below sustainable transmission levels. This is equivalent to tolerating that MDA will start or continue in 10% of areas that do not require it (overtreatment; Type II error = 10%).

                              ⇩

**3. Calculate required specificity**

Calculations indicate that to ensure a < 10% Type II error,

a survey must be conducted using a ≥ 99.8% specific assay.

                              ⇩

**4. Establish acceptable Type I error**

Accept that 5% of surveys will suggest that onchocerciasis transmission is not occurring, when in fact, it is. This is equivalent to tolerating that MDA will not be initiated or will be stopped in 5% of areas that require it (undertreatment, Type I error = 5%).

                              ⇩

**5. Calculate required sensitivity**

**5.1 Mapping**

Calculations indicate that to detect a 2% prevalence with a 99.8% specific test, and ensure a < 5% Type I error,

- survey must be conducted using a ≥ 60% sensitive assay.

**5.2 Stopping**

Calculations indicate that to detect a 1% prevalence with a 99.8% specific test, and ensure a < 5% Type I error, a survey must be conducted using a ≥ 89% sensitive assay.

                              ⇩

**Summary**

For mapping the assay must be ≥ 60% sensitive and ≥ 99.8% specific.

For stopping the assay must be ≥ 89% sensitive and ≥ 99.8% specific.

To support decision-making based on the average prevalence in the survey area, assuming a sample size of 3000 children, a specificity of 99.6% is required to secure a Type II error < 10% in non-endemic areas (**Table 5**). With this specificity, a sensitivity of 50% suffices to detect 1% of prevalence with a Type I error < 5% (**Table 6**). For the more stringent decision criteria, whereby the results from each cluster are compared independently to a critical cutoff, a specificity of 99.8% and a sensitivity of 89% are required to meet a 10% Type II error and a 5% Type I error (**Tables 7 and 8**). The critical cutoff for these cluster-level decisions was set at ≥ 3 positives per village.

**Table 5:** Effect of specificity on the Type II error of a 30-cluster survey, where treatment decisions are based on the average prevalence of the entire survey area. No sensitivity assumptions were made. The critical cutoff was ≥ 19 positive tests out of a sample size of 3000. The calculations were made for a true prevalence of 0%. At least 99.6% specificity is required to have a Type II error of < 10%.

Entry	Sensitivity	Specificity	True Prevalence	Critical cutoff	Type II error
1	NA	99.5%	0%	≥ 19 positives	18.0%
2	NA	99.6%	0%	≥ 19 positives	3.7%
3	NA	99.7%	0%	≥ 19 positives	0.2%

**Table 6:** Effect of sensitivity on the Type I error of a 30-cluster survey involving 3000 children, where treatment decisions are based on the average prevalence of the entire survey area. A specificity of 99.8% was assumed. The critical cutoff was ≥ 19 positive tests. The calculations were made for a true prevalence of 1%. At least 50% sensitivity is required to have a Type I error of < 5%.

Entry	Sensitivity	Specificity	True Prevalence	Critical cutoff	Type I error
1	100%	99.6%	1%	≥ 19 positives	0%
2	90%	99.6%	1%	≥ 19 positives	0.01%
3	80%	99.6%	1%	≥ 19 positives	0.07%
4	70%	99.6%	1%	≥ 19 positives	0.33%
5	60%	99.6%	1%	≥ 19 positives	1.3%
6	50%	99.6%	1%	≥ 19 positives	4.58%

**Table 7:** Effect of specificity on the Type II error when treatment decisions are based on the cluster-specific results, assuming 30 clusters are surveyed and 100 children/cluster. No sensitivity assumptions were made. The critical cutoff was ≥ 3 positive tests in at least 1 village. The calculations were made for a true prevalence of 0%. At least 99.7% specificity is required to have a Type II error of < 10%.

Entry	Sensitivity	Specificity	True Prevalence	Critical cutoff	Type II error
1	NA	99%	0%	≥ 3 positives	91.6%
2	NA	99.5%	0%	≥ 3 positives	34.7%
3	NA	99.6%	0%	≥ 3 positives	20.8%
4	NA	99.7%	0%	≥ 3 positives	10.1%
5	NA	99.8%	0%	≥ 3 positives	3.3%
6	NA	99.9%	0%	≥ 3 positives	0.5%

**Table 8:** Effect of sensitivity on the Type I error when treatment decisions are based on the cluster-specific results, assuming 30 clusters are surveyed and 100 children/cluster. A specificity of 99.8% was assumed. The critical cutoff was ≥ 2 positive tests in at least 1 village. The calculations were made for a true prevalence of 1%. At least 89% sensitivity is required to have a Type I error of < 5%.

Entry	Sensitivity	Specificity	True Prevalence	Critical cutoff	Type I error
1	100%	99.8%	1%	≥ 3 positives	2.2%
2	95%	99.8%	1%	≥ 3 positives	3.2%
3	90%	99.8%	1%	≥ 3 positives	4.5%
4	89%	99.8%	1%	≥ 3 positives	4.8%
5	88%	99.8%	1%	≥ 3 positives	5.2%

In the absence of a definitive strategy, the subgroup recommended the parameters for decision-making based on cluster-level results, which provides the highest standard, namely 89% sensitivity and 99.8% specificity.

## Discussion

Several of the criteria listed in the TPPs are not surprising for a test aimed at supporting an NTD program: an ideal test for onchocerciasis needs to be simple to operate under zero infrastructure, be heat-stable, and be inexpensive. It was also noted that laboratory tests could be acceptable, despite the lack of infrastructure in many countries, since the current serological and entomological techniques already require laboratories.

One challenge in the field of onchocerciasis is that a biomarker indicative of the presence of live female *O*. *volvulus* worms, while ideal, has remained elusive. To strike a balance between ideal and pragmatic scenarios, the TPPs accepts serology tests which, even if not indicative of active infections, are likely to be more rapidly developed.

Perhaps the most important parameters that guide any test development are the sensitivity and specificity. The target product profiles presented herein both require a strikingly high 99.8% specificity. The sensitivity should exceed either 60% (mapping) or 89% (stopping). The subgroup recognizes that developing a test and demonstrating a 99.8% specificity will be challenging. One issue is that no test can be considered the gold standard. A second issue is that it will be necessary to gather data on thousands of people and that field validation studies and their statistical analysis will require careful design in appropriate settings.

The sensitivity and specificity requirements were produced using the best available information regarding biological thresholds to be detected for onchocerciasis elimination mapping and for supporting mass drug administration cessation decisions. Calculations assumed that one needs to detect a 1–2% prevalence of the biomarker under scrutiny, and this was based on data for Ov16-specific antibodies, but these biological thresholds may change in the future based on new empirical evidence and modelling studies. In addition, the prevalence of Ov-16 specific antibodies, a marker of exposure, is known to exceed the prevalence of skin microfilariae, a marker of active infection [37]. This distinction was not considered while developing the TPPs, and future refinements of the TPPs may introduce different biological thresholds for antibody- versus antigen-detection.

## Conclusions

In conclusion, the most demanding requirement of tests for onchocerciasis will be to demonstrate a specificity of > 99.8%. In addition, the sensitivity should exceed either 60% (Onchocerciasis Elimination Mapping to start MDA) or 89% (stop MDA). All other requirements are what could be expected for a test that will support a Neglected Tropical Disease, including being preferably field-deployable, cost-effective, and compatible with tropical climates. As per WHO guidelines, the TPPs presented should be reviewed every 5 years, unless new data prompt a faster review.

Regarding future work, the subcommittee also examined suggestions to develop TPPs for (1) monitoring and evaluation, (2) entomology, and (3) defining transmission zones. Given that the needs and vector biology (species, density, biting rate) differ widely from place to place, our subgroup believes it will be best to let the monitoring and evaluation strategy be handled by national onchocerciasis committees, and therefore does not recommend developing a TPP for this use case at this time. For entomological evaluations, it is recognized that the O-150 PCR test, being one of the two methods recommended in the WHO 2016 Guidelines for Stopping MDA and Verifying Elimination of Human Onchocerciasis [30], is especially relevant to programs and that there is a need for quality assured, PCR reagents that would not require a cold chain (*e*.*g*., customs delays). Furthermore, both PCR and ELISA methods need a formal, standardized protocol that can be easily adopted in multiple laboratories. This topic represents a program issue that will be discussed further. As for defining transmission zones, this effort was deemed premature given the current scientific knowledge. Finally, though technically not a TPP, it was recognized that it would be extremely useful to have a way to tie current infection rates determined by serology to historic, baseline prevalence rates determined by skin biopsies or nodule palpation.
