# Correction: Onchocerciasis: Target product profiles of in vitro diagnostics to support onchocerciasis elimination mapping and mass drug administration stopping decisions

**DOI:** 10.1371/journal.pntd.0011505

**Published:** 2023-07-19

**Authors:** Marco A. Biamonte, Paul T. Cantey, Yaya I. Coulibaly, Katherine M. Gass, Louise C. Hamill, Christopher Hanna, Patrick J. Lammie, Joseph Kamgno, Thomas B. Nutman, David W. Oguttu, Dieudonné P. Sankara, Wilma A. Stolk, Thomas R. Unnasch

Point 5 of the section ‘Available diagnostic tools and their limitations’ incorrectly states that the BIOLINE Onchocerciasis IgG4 ELISA (Abbott) is discontinued. As of July 2023, the ELISA kit is still available, catalog number 61EK11.
